# When the nerve speaks first: IgG4-related disease unmasked by peripheral neuropathy

**DOI:** 10.3389/fneur.2026.1776740

**Published:** 2026-02-19

**Authors:** Yi Zhou, Xinran Ma, Jinxia Zhao, Kun Wang, Danfeng Zheng, Hongsong Song, Dongsheng Fan

**Affiliations:** 1 Department of Neurology, Peking University Third Hospital, Beijing, China; 2 Department of Neurology, The Second Affiliated Hospital of Chengdu Medical College, China National Nuclear Corporation 416 Hospital, Chengdu, China; 3 Beijing Key Laboratory of Biomarker and Translational Research in Neurodegenerative Diseases, Beijing, China; 4 Key Laboratory for Neuroscience, National Health Commission/Ministry of Education, Peking University, Beijing, China; 5 Department of Rheumatology and Immunology, Peking University Third Hospital, Beijing, China; 6 Department of Gastroenterology, Peking University Third Hospital, Beijing, China; 7 Department of Pathology, School of Basic Medical Sciences, Peking University Third Hospital, Peking University Health Science Center, Beijing, China

**Keywords:** corticosteroid therapy, demyelinating polyneuropathy, elevated serum IgG4, immunoglobulin G4-related disease, mononeuritis multiplex, multi-organ involvement, peripheral neuropathy

## Abstract

**Background:**

IgG4-related disease (Immunoglobulin G4-related disease) is an immune-mediated condition characterized by elevated serum IgG4 levels, clinically presenting as multi-organ enlargement. However, only case reports exist of IgG4-related disease presenting initially with peripheral neuropathy. This study aims to elucidate the clinical features of IgG4-related disease with prominent peripheral neuropathy manifestations.

**Methods:**

We reviewed 16 cases of IgG4-related disease with peripheral neuropathy as the initial presentation, including 15 cases from the literature and one case from our institution. Patient data were extracted and analyzed, encompassing clinical characteristics, pathological features, electrophysiological findings, and treatment information.

**Results:**

The median age of onset was 64.5 years, with 3 female and 13 male patients. Initially, 15 patients presented with limb numbness and weakness, while 1 exhibited hoarseness. All 16 patients demonstrated organ enlargement in addition to peripheral nerve damage. Peripheral nerve electrophysiology revealed demyelination and/or axonal lesions. All cases received oral corticosteroids, with immunosuppressants added in 3 cases. All patients responded well to corticosteroid therapy.

**Conclusion:**

Peripheral neuropathy may present as the initial manifestation of IgG4-related disease. Such neuropathy demonstrates favorable response to corticosteroid therapy, and the indication for adding immunosuppressive agents should be evaluated based on individual circumstances.

## 1 Introduction

Immunoglobulin G4-related disease (IgG4-RD) is an immune-mediated fibro-inflammatory disease. Its primary pathological features include lymphoplasmacytic infiltration dominated by IgG4-positive plasma cells, accompanied by dendritic fibrosis, occlusive phlebitis, and eosinophilic infiltration. This disease can affect multiple organs, including the pancreas, salivary glands, kidneys, and arteries (1). Immunoglobulin G4–related disease (IgG4-RD) is a systemic immune-mediated fibroinflammatory disease. Since its discovery nearly two decades ago, our understanding of its pathophysiology and clinical manifestations has grown substantially. Early diagnosis and treatment of this elusive disease can prevent substantial organ damage from end-stage fibrosis, emphasizing the need for prompt recognition and accurate characterization of IgG4-RD. The classification criteria endorsed by the American College of Rheumatology and the European Alliance of Associations for Rheumatology in 2019 provide a framework for establishing the diagnosis in the clinical setting. This process involves recognizing the typical manifestations of the disease and incorporating clinical, radiological, serological, and histopathological information as well as excluding disease mimickers. Glucocorticoids and rituximab are effective at inducing remission in IgG4-RD in most patients, but the optimal approach to long-term management of IgG4-RD remains an area of active clinical research. Previous studies have documented neurological involvement, such as IgG4-related hypophysitis and proliferative meningitis (2). However, IgG4-related disease presenting with peripheral neuropathy remains extremely rare in the literature. Primary objective was to delineate the clinical–electrophysiological–pathological phenotype; secondary objectives were to propose diagnostic clues and to assess treatment response.

## 2 Methods

### 2.1 Literature review

We conducted a literature review to identify cases of IgG4-related disease primarily manifesting as peripheral neuropathy. In this study, IgG4-related peripheral neuropathy was defined as nodular or enlarged lesions in one or more organs, elevated serum IgG4 levels (normal range: less than135 mg/dL), and histopathological findings meeting two of the following three criteria: (1) occlusive phlebitis or tissue fibrosis; (2) IgG4-positive plasma cells/IgG-positive plasma cells constituting at least 40% of cells; (3) dense lymphocytic and plasma cell infiltration with fibrosis, with peripheral neuropathy as the predominant clinical manifestation. A comprehensive literature search was conducted in PubMed/Medline and Chinese databases from inception to November 30, 2025. The search strategy employed a combination of Medical Subject Headings (MeSH) terms and free-text terms. In English databases, we used “Immunoglobulin G4-Related Disease,” “Immunoglobulin G4 Related Disease,” “Immunoglobulin G4-Related Diseases,” “IgG4-Associated Autoimmune Disease,” “Autoimmune Disease, IgG4-Associated,” “IgG4 Associated Autoimmune Disease,” IgG4-Associated Autoimmune Diseases,““IgG4-Related Disease,““IgG4 Related Disease,““IgG4-Related Diseases,““IgG4-RD, “or” IgG4 Related Systemic Disease. “These terms were combined with” Peripheral Nervous System Diseases,““Peripheral Nervous System Disorders,““Peripheral Neuropathies,““Neuropathy, Peripheral,““Peripheral Neuropathy,““Peripheral Nervous System Disease,““PNS Diseases,““PNS Disease,““Peripheral Nerve Diseases,““Nerve Disease, Peripheral,““Nerve Diseases, Peripheral,““Peripheral Nerve Disease, “or” PNS (Peripheral Nervous System) Diseases.” Manually search the references of identified articles to identify additional relevant studies.

### 2.2 Case selection

This study followed the 2019 ACR/EULAR classification criteria. A two-step inclusion algorithm was applied: first, objective evidence of peripheral nerve injury (clinical symptoms plus electrophysiological or imaging confirmation) was required; second, patients had to meet ‘definite’ or ‘probable’ IgG4-related disease (IgG4-RD) categories. When nerve biopsy was not feasible, a typical biopsy from an adjacent affected organ was acceptable, provided that serum IgG4 ≥ 135 mg/dL and characteristic imaging findings were simultaneously present. Exclusion criteria encompassed peripheral neuropathies attributable to diabetes, vasculitis, paraneoplastic disorders, infection, malignancy, and histological features inconsistent with IgG4-RD (prominent neutrophilic infiltrates, necrotizing vasculitis, etc.). For literature cases, the original report had to describe both neurological manifestations and sufficient IgG4-RD diagnostic details; Considering IgG4-related eye disease as a classification of IgG4-related disease, we excluded IgG4-related cranial nerve damage from this study. Cases were reviewed by two independent reviewers, with discrepancies resolved through consensus or consultation with a third reviewer. Additionally, data were extracted from selected cases using standardized forms via retrospective review of literature cases, two reviews indecently extracted data using a predefined 14-item form (Supplementary File S1). Limitations of our review methodology, including the retrospective nature of the included studies and potential publication bias, were explicitly considered during analysis and interpretation of findings. Consequently, 16 patients were ultimately selected for inclusion in the study.

## 3 Results

### 3.1 Patients profiles

Among the 16 selected patients (including one typical case from our hospital), 13 were male and 3 were female. The age of onset was 63.8 ± 13.1; except for one patient aged 31, all others were over 50 years old at onset. The patient’s serum IgG4 level was 542.0 (262.0–736.7) mg/L, significantly higher than the normal value (135 mg/dL), and all cases were pathologically confirmed. The sampling sites and pathological results for each case have been added to Supplementary Table 1.

### 3.2 Multiple organ involvement

All enrolled patients exhibited multi-organ involvement, primarily manifested as enlargement of organs or tissues. Among these, 3 cases presented renal masses or perirenal changes, 2 cases showed pancreatic enlargement, 5 cases had lymphadenopathy, 1 case exhibited periorbital inflammatory pseudo-tumor, 2 cases demonstrated skin induration or hyperpigmentation, 1 case had a pleural mass, 1 case showed a periaortic mass, and 3 cases presented swelling of the submandibular gland, thyroid gland, or salivary glands.

### 3.3 Manifestations of peripheral neuropathy

All cases exhibited peripheral nerve involvement, presenting with varying degrees of limb numbness and weakness. The characteristics of peripheral nerve damage can be broadly categorized into three types. The first category primarily manifested as axonal peripheral neuropathy. Based on retrospective case retrieval, this constituted the largest proportion. It could be further subdivided into two subtypes: vasculitis-mediated mononeuropathy (predominant) and non-vasculitis-mediated mechanisms. Regarding the vasculitis-mediated mechanism, nerve biopsies revealed IgG4 + plasma cell infiltration and epithelial fibrosis. This likely resulted from inflammatory cell infiltration and perineural fibrosis obstructing blood vessels, causing peripheral nerve ischemia and subsequent axonal lesions. The second category presents as compressive peripheral neuropathy, likely associated with the mass effect of lesions in adjacent organs. The third category manifests as chronic inflammatory demyelinating polyneuropathy (CIDP).

### 3.4 Treatment response

All cases received oral or intravenous steroid therapy, primarily with prednisone and methylprednisolone. Symptoms improved significantly in all patients following steroid administration, with only one case experiencing residual mild limb numbness. Three cases additionally received immunosuppressive agents, primarily mycophenolate mofetil, methotrexate, and rituximab.

### 3.5 Representative cases from our institution

#### 3.5.1 Case no. 12 (our case)

A 63-year-old male patient was admitted complaining of progressive weakness in both lower limbs lasting over 3 months, accompanied by persistent abnormal sensations in both hands and feet for over one month. Symptoms began approximately 3 months prior to admission, when the patient experienced unexplained weakness in both lower limbs. He reported difficulty climbing stairs and rising from a squatting position, though he could still walk on level ground. Symptoms gradually worsened until he could no longer independently rise from a squatting position, and he noted marked muscle wasting in both thighs. The patient denied experiencing myotonic tremors, dyspnea, or diurnal fluctuations in symptom severity. He also reported no limb pain, bowel or bladder dysfunction, or abnormal nocturnal behavior. The patient initially sought care at a local hospital, where lower limb electromyography (EMG) revealed “demyelinating lesions with axonal damage and abnormalities in deep sensory conduction pathways.” No specific treatment was initiated at that time. Then, the patient was referred to our hospital’s neurology outpatient clinic. Given the long-term history of alcohol consumption, the preliminary diagnosis leaned toward “alcoholic peripheral neuropathy.” Symptomatic treatment was initiated with oral vitamin B12, vitamin B1, and folic acid.

Patient had a 10-year history of hypertension and was intermittently treated with amlodipine 5 mg once daily. He stopped the medication 3 months ago and his blood pressure has remained within normal range since then. Neurologic examination revealed bilateral quadriceps atrophy. Muscle strength testing showed proximal 4/5 (Medical Research Council Rating Scale) in the lower extremities, 1/5 in the tibialis anterior bilaterally, and 4/5 in the gastrocnemius bilaterally. Upper-extremity tendon reflexes were diminished, and no tendon reflexes were observed in the lower extremities. Vibratory sensation was diminished in the left lower extremity and Romberg’s sign was positive. Cranial nerves, as well as sensory, motor, coordination, and tendon reflexes of the upper limbs, are all normal. A detailed neurological examination can be seen in Supplementary Table 2.

#### 3.5.2 Routine blood test and other examinations

Serum immunofixation electrophoresis revealed an elevated α2-globulin level at 10.2%. Lambda light chains were also elevated at 663 IU/mL. Antinuclear antibodies (ANA) showed a speckled pattern with a titer of 1:80, and anti-RNP/Sm antibodies were weakly positive. Tumor markers were mildly elevated, including squamous cell carcinoma antigen at 3 mg/mL and cytokeratin fragment CYFRA 21-1 at 5.42 ng/dL. Serum lipase and amylase levels were slightly elevated, with lipase at 328 U/L and amylase at 115 U/L. Cerebrospinal fluid (CSF) pressure was 110 mmH₂O, and CSF analysis showed a normal cell count with mildly elevated total protein (232 mg/dL). Serum IgG4 levels were elevated at 3.85 g/L. Blood and cerebrospinal fluid samples revealed no abnormalities in rheumatoid factor, Anti-NF155 (Neurofascin-155) antibody, Anti-NF186 (Neurofascin-186) antibody, human immunodeficiency virus, syphilis antibodies, or malignancies.

**Figure 1:**
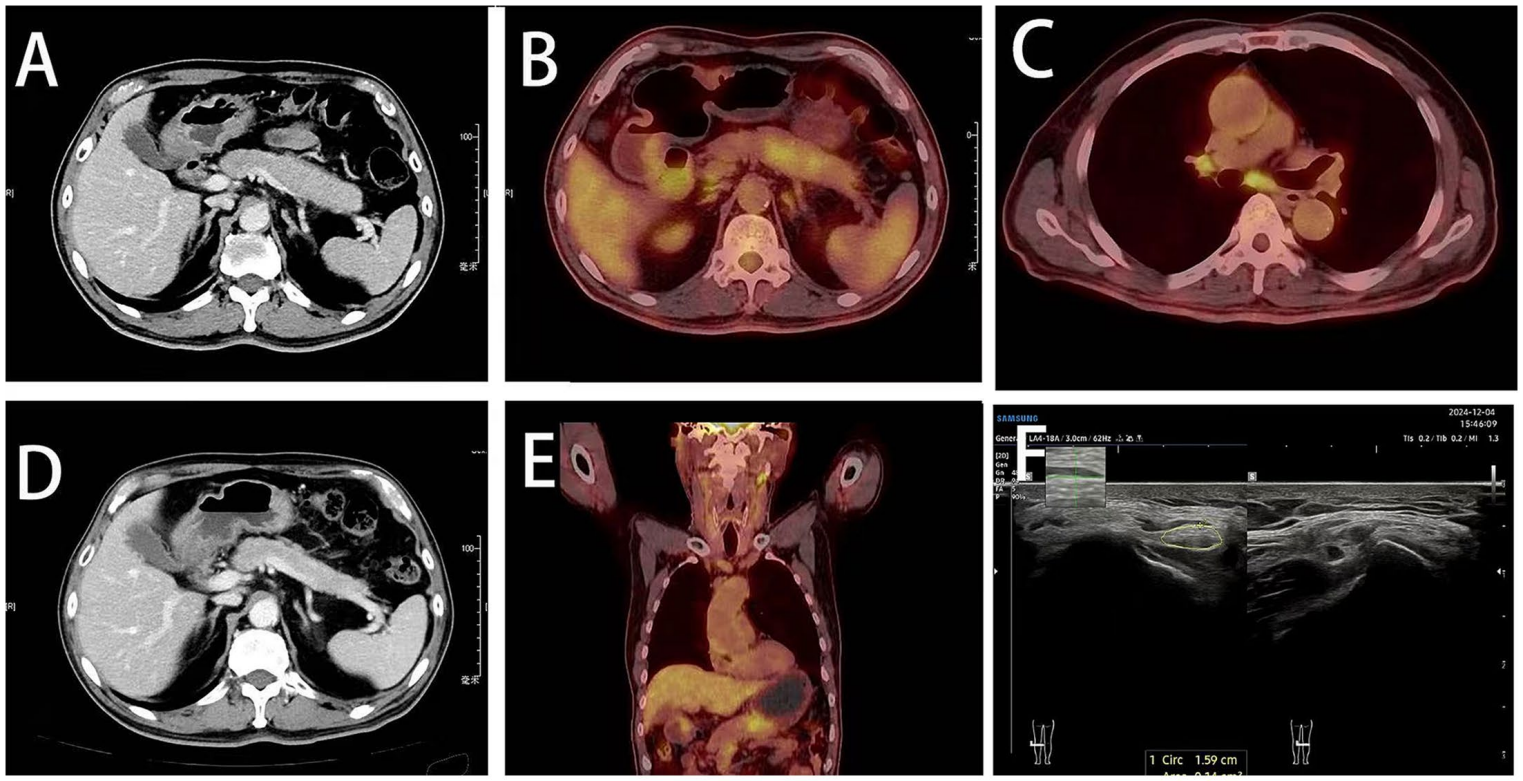
Radiographic features. **(A)** Enlarged body and tail of the pancreas visualized on abdominal CT (pre-steroid therapy). **(B)** PET-CT showed enlarged body and tail of the pancreas. **(C)** Enlarged lymph nodes in mediastinal zones 4R and 7 on PET-CT (pre-steroid therapy). **(D)** Abdominal CT showed pancreas with decreased swelling after treatment. **(E)** PET-CT showed swollen lymph nodes in the neck (pre-steroid therapy). **(F)** Lower limb nerve ultrasound reveals several nerve bundles of the right common peroneal nerve are enlarged (before steroid use).

Ultrasound of the right common peroneal nerve showed mild swelling near the head of the fibula (Figure 1F). Carotid ultrasound showed the presence of bilateral carotid atherosclerotic plaques. Thyroid ultrasound showed nodular goiter. Salivary gland ultrasound revealed heterogeneous echogenicity of submandibular and sublingual glands. Abdominal CT showed enlargement of the body and tail of the pancreas suggestive of autoimmune pancreatitis (Figure 1A). The same imaging features of the pancreas were observed on PET-CT (Figure 1B). Multiple lymph nodes was also observed on PET-CT (Figures 1C,E). Chest CT presented few ground glass nodules in both lungs. No significant abnormalities were detected in the rest of the peripheral nerve ultrasound, brain MRI, cervical spine MRI or biliary tract.

#### 3.5.3 Nerve and submandibular gland biopsy

**Figure 2:**
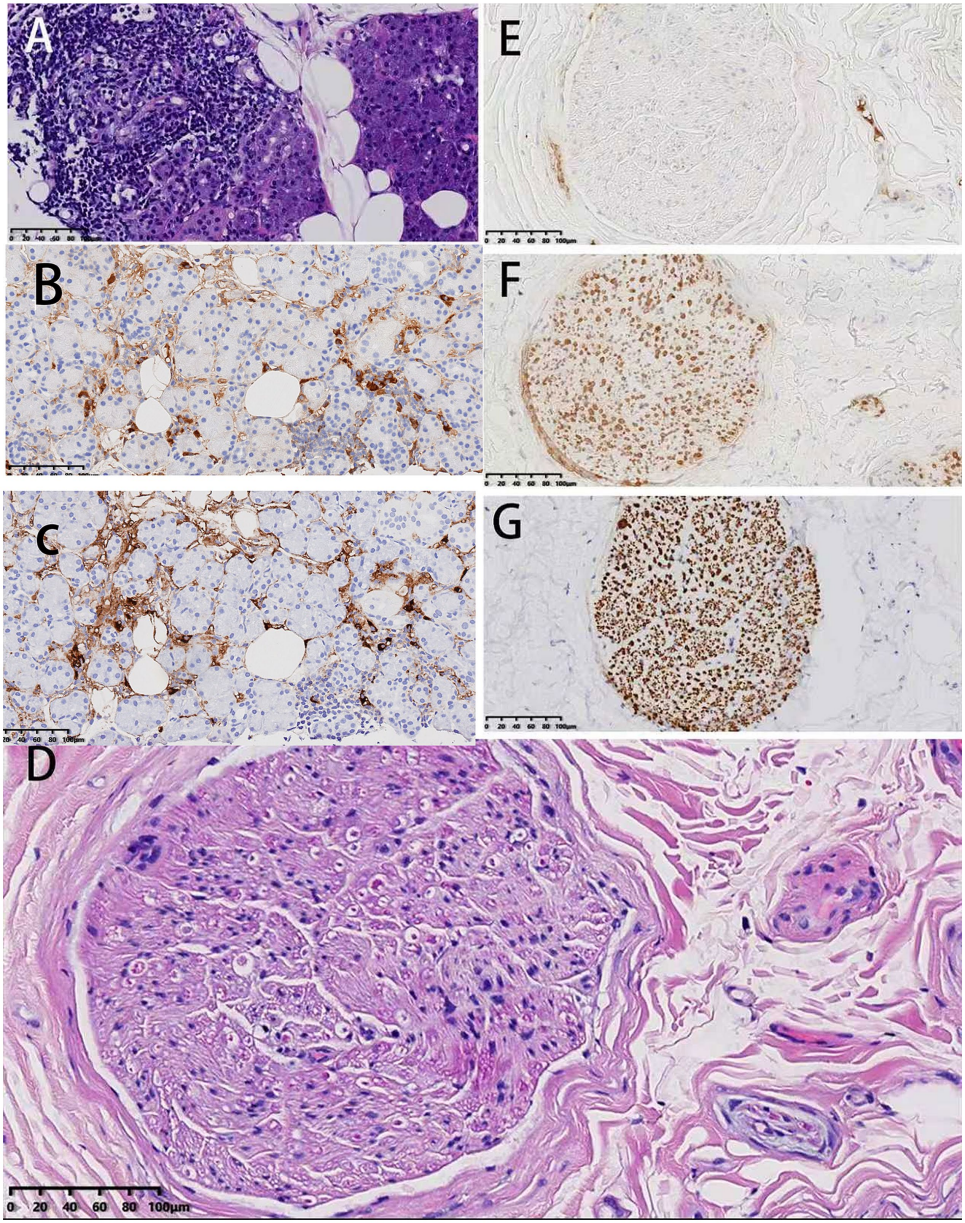
Pathology results for patients at our hospital (case 12). **(A–C)** Pathology of right submandibular gland: **(A)** Hematoxylin and eosin (HE) staining, 20×; **(B)** IgG staining, 20×; **(C)** IgG4 staining, 20×. Focal mild glandular atrophy with three lymphocytic foci visible. No significant fibrosis or vasculitis observed. IgG4/IgG-positive plasma cells >50%. **(D–G)** Sural nerve pathology: **(D)** HE 20×, **(E)** IgG4 20×, **(F)** MBP 20×, **(G)** NF 20×. Immunohistochemical staining revealed an absence of significant IgG4-positive plasma cell infiltration in the nerve biopsy samples.

Biopsy of the right submandibular gland was suggestive of IgG4/IgG positive plasma cells >50%, with no significant fibrosis or vasculitis. Immunohistochemistry was suggestive of IgG (+), IgG4 (10/HPF), CD3 (T-cells +), CD 20 (B-cells +), CD138 (scattered +) (Figure 2). Pathological findings of left sural nerve biopsy showed a decrease in myelinated fibers, edematous degeneration of individual intra-axonal organelles, partially accompanied by myelin loosening, a fair number of unmyelinated fibers, no obvious Schwann cell proliferation, and no obvious amyloid deposition. Immunohistochemistry was positive for CD 68, CD 20 and CD 138 (Figure 2).

#### 3.5.4 Nerve conduction studies and electromyography

Electromyographic studies were performed to measure motor nerve conduction in the bilateral median, ulnar, tibial, and common peroneal nerves. Compound muscle action potentials (CMAP) and motor conduction velocity (MCV) were decreased. Sensory conduction velocity (SCV) was not detected bilaterally in the ulnar and superficial peroneal nerves. Myelin and axonal damage were present (Table 1).

**Table 1:** Results of nerve conduction study and needle electromyography.

NCS	Right/Left			NCS	Right/Left		Muscle	Activity		Mean Amp		Mean dur	Recruitment phase
	Latency	Amp	NCV		Amp	NCV		Fib	PSW	uV	Ref. Dev		
	(ms)	(mv)	(m/s)		(mv)	(m/s)							
Motor NCS				Sensory NCS									
Median nerve				Median nerve			Right						
Wrist-Palm	5.6/5.03	5.1/4.7	28.1/30.6	Dig I-wrist	NE/3.9	NE/32.7	Ext dig communis	−	−	778	Normal	Normal	Simple mixed
Elbow-wrist	13.4/11.7	3.0/3.1	29.5/34.5				Sternocleidomastoid	−	−	643	Normal	Normal	Simple mixed
Axilla-elbow	20.3/16.7	1.17/1.95	17.4/20.0				Tibialis anterior	+++	+++	1,126	Increased 138%	Normal	Simple
							Left						
Ulnar nerve				Ulnar nerve			Gastrocnemius	+++	+++	1,138	Increased 255.6%	Normal	
Wrist-ADM	3.71/4.64	3.4/4.1		Dig V-wrist	NE/NE	NE/NE	Inteross dors I	++	++	752	Normal	Normal	Simple
Below elbow-wrist	10.6/10.3	3.4/4.2	27.6/33.5				Rectus abdominis	−	−	383	Normal	Normal	Simple mixed
Above elbow-below elbow	14.1/13.3	3.2/4.6	28.6/33.3										
Axilla-Above elbow	17.5/18.7	1.32/1.76	26.5/18.5										
Peroneus				Superficial peroneal nerve	NE/NE	NE/NE							
Ankle-EDB	7.5/10.6	0.52/0.12											
Fib up-Ankle	31.2/37.2	0.078/0.12											
													
Tibialis				Sural nerve	NE/6.5	NE/31.2							
Ankle-Abd hal	8.38/8.1	1.72/1.35											
Knee-ankle	26.7/26.4	0.69/0.72	21.3/20.8										

Amp, amplitude; NCV, nerve conduction velocity; ADM, abductor digiti minimi; EDB, extensor digitorum brevis; Fib up, fibularis longus; Abd hal, abductor hallucis; Dig I, digastricus I; Dig V, digastricus V; dur, duration; Ext dig, extensor digitorum communis; Inteross dors I, first dorsal interosseous; Fib, fibrillation potentials; PSW, positive sharp waves; NE, Not Evoked.

#### 3.5.5 Treatment and follow-up

Oral glucocorticoid therapy (prednisolone, 60 mg/day for 4 weeks, followed by weekly tapering of 5 mg) was administered concurrently with mycophenolate mofetil 1 g twice daily. At the 3-month follow-up, serum IgG4 levels decreased (from 385 mg/dL to 56 mg/dL), and abdominal CT showed the enlarged pancreas gradually returning to normal size (Figure 1D). Weakness in both lower limbs and bilateral numbness in both hands completely resolved, with only residual numbness in the soles of both feet. Notably, during recovery, the patient experienced episodes of diplopia and left-sided facial numbness, which resolved completely within 4–5 days. Specific symptoms during these episodes remain unclear as the patient did not seek medical attention at the time. Subsequently, the patient was maintained on a long-term low-dose corticosteroid regimen of 5 mg daily, with no recurrence of limb numbness, weakness, or gait instability.

## 4 Discussion

This retrospective study analyzed the clinical manifestations, electrophysiological findings, and treatment responses in 16 cases of IgG4-related disease primarily affecting peripheral nerves. A detailed clinical course description is provided for one patient from our institution. Among the 16 reviewed cases, males predominated (13 cases), with a mean age of 63.75 years (Table 2). As noted in Abdelrazek MA’s review (3), the typical IgG4-related disease patient is a middle-aged or elderly male, with a mean age at diagnosis ranging from 55 to 59 years and a male-to-female ratio of 1:0.64 to 1:0.77. Disease severity shows no significant correlation with gender (4, 5).

**Table 2:** Clinical characteristics, serum IgG4 level, and initial medication.

No.	Age/Sex	Neurological symptoms	Neuropathic manifestations	Other system damage	Serum IgG4 (mg/dL)	Initial dose of steroid	Immuno-suppressants	Reference
1	69/F	Right-hand weakness	Mononeuropathy multiplex,axonal injury	Renal mass	177.8	0.6 mg/kg/d IVIG*10 day, 0 mg/d	Methotrexate	(13)
2	81/F	Unilateral ptosis, eyelid swelling, abnormal sole sensation	Mononeuropathy multiplex	Sinusitis, thyroiditis, sialadenitis	1,310	10 mg/d	None	(14)
3	69/M	Numbness in hands and feet, distal lower limb weakness	Mononeuropathy multiplex,axonal injury	Lymphadenopathy	712	0.5 mg/kg/d	None	(14)
4	65/M	Limb numbness and weakness	Mononeuropathy multiplex,axonal injury	Pancreatic swelling	1,660	80 mg/d	None	(15)
5	55/F	Right lower limb weaknessinitially, progressing to bilateral leg weakness, numbness, and pain	Mononeuropathy multiplex,axonal injury	Renal enlargement	177	40 mg/d	None	(16)
6	55/M	Lower limb pain and numbness, bilateral hand weakness	Mononeuropathy multiplex, axonal injury	Skin induration and pigmentation	259	30 mg/d	Rituximab	(17)
7	56/M	Sudden asymmetric onset of limb numbness and weakness	Mononeuropathy multiplex, axonal injury	Cutaneous pigmentation, edema, lymphadenopathy, hepatosplenomegaly	328	40 mg/d	None	(18)
8	82/M	Recurrent limb numbness and weakness	Mononeuropathy multiplex, slowed nerve conduction velocity	Edema, upper eyelid swelling, salivary gland swelling, arthritis	630	0.6 mg/kg/d	None	(19)
9	77/M	Lower limb numbness and weakness	Lower Limb Motor Nerve Impairment	Interstitial lung disease, mediastinal lymphadenopathy, proteinuria	728	1 g IVIG*3 day	None	(20)
10	73/M	Rapidly progressing neuropathy, lower limb discomfort, numbness and weakness	Severe length-dependent axonal neuropathy with right L5 radiculopathy and right ulnar neuropathy	Pleural mass	454	40 mg/d	None	(8)
11	31/M	Hoarseness	Recurrent laryngeal nerve palsy lowed nerve conduction velocity	Periaortic mass	263	80 mg/d*14 day, 40 mg/d	None	(7)
12	63/M	Weakness in both lower limbs, limb numbness	CIDP-like manifestations	Pancreatic enlargement, submandibular gland swelling	385	60 mg/d	MycophenolateMofetil	Present case
13	55/M	Weakness and numbness in all four limbs	CIDP-like manifestations	Orbital pseudotumor, left optic nerve and extraocular muscle enlargement	1,550	NA	None	(1)
14	64/M	Weakness and sensory disturbance in the left upper limb	Normal MCV, MRN showing multiple peripheral nerve swellings	Lymphadenopathy	762.7	40 mg/d	None	(21)
15	74/M	Numbness and weakness starting in the distal lower limbs	Multiple radiculoneuropathy, demyelination with secondary axonal damage, compression at the fibular head and cubital tunnel	Pleural effusion, myopathy, lymphadenopathy, dry mouth and eyes	217	30 mg/d	None	(22)
16	51/M	Numbness in the right lower limb	NA	Dural hyperplasia, perirenal lesions	724	30 mg/d	None	(23)

IgG4-related disease can involve multiple organs and may present as organ enlargement. In the 16 patients in this study, evidence of at least one or more organ involvement was observed in addition to symptoms of peripheral nerve damage. In summary, when IgG4-related disease is suspected, multi-organ screening provides stronger diagnostic support, particularly for the pancreas, lacrimal glands, submandibular glands, and kidneys. As demonstrated in this case, the discovery of pancreatic enlargement raised suspicion of IgG4-related disease and further guided physicians toward an accurate diagnosis.

Peripheral nerves may be involved in IgG4-related disease through one of three mechanisms. First, the local mass effect of non-neural tissue may compress nerves (6). A case report from Japan demonstrated that a periarterial mass caused recurrent laryngeal nerve palsy (7). Additionally, another prominent manifestation of IgG4-related disease affecting the nervous system is IgG4-related orbital disease, which is also partially caused by mass effect. In a cohort of 172 patients with IgG4-related orbital disease, 68% presented with bilateral lesions, primarily manifesting as eyelid swelling, exophthalmos, diplopia, and visual acuity decline, partially attributable to orbital mass effect (8). Second, peripheral nerves are affected by the chronic, persistent inflammatory aggregation response characteristic of IgG4-related disease (Case 7, 8). Infiltration of inflammatory cells and fibrosis of the perineurium lead to vascular obstruction, ischemia of the perineurium, vasculitic pathological changes, and ultimately axonal lesions. Several patients reviewed in this study appear to favor this mechanism (case 3, 4, 7, 8), which is also the most common pathological mechanism in the literature review. A retrospective study of 149 patients previously diagnosed with various inflammatory neuropathies via nerve biopsy revealed that 29 patients (11 diagnosed with vasculitic neuropathy) met IgG4-related disease criteria upon IgG4-related disease staining (9). The mechanism of axonal injury may be associated with functional nutritional alterations induced by chronic inflammation. Finally, IgG4 antibodies disrupt the ganglion-adjacent adhesion complex, leading to dysfunction of the ganglion-adjacent structures. This results in non-inflammatory, reversible conduction block, producing CIDP-like changes. Our institution favors this mechanism for the patient presented. In our institution, the patient’s nerve biopsy did not show significant IgG4-positive plasma cells, but the submandibular gland biopsy indicated that the proportion of IgG4-positive plasma cells to IgG-positive plasma cells was greater than 40%. Considering the patient has pancreatic enlargement, elevated serum IgG4 levels, and responds well to steroids, we believe it is reasonable to diagnose IgG4-related disease-associated peripheral neuropathy. In summary, the mechanisms underlying peripheral neuropathy in IgG4-related disease are local mass effects of non-neural tissues, chronic inflammation, and perilesional functional impairment.

Regarding laboratory testing, both serology and cerebrospinal fluid analysis are equally crucial for diagnosing IgG4-related peripheral neuropathy. While significantly elevated serum IgG4 levels serve as a favorable indicator for IgG4-related diseases, these are insufficient for definitive diagnosis alone. One meta-analysis of 1,200 IgG4-related disease patients and 5,700 healthy controls demonstrated that using serum IgG4 levels >135 mg/dL as a diagnostic criterion yielded a sensitivity of 87% and specificity of 83% (10). Elevated serum IgG4 levels were observed in all 16 patients reviewed in this study, providing diagnostic support for IgG4-related disease. Consequently, elevated serum IgG4 levels should alert clinicians to the potential presence of this disease in other organ systems. Given that steroid significantly reduce serum IgG4 levels, the optimal timing for IgG4 measurement is prior to steroid administration (11). IgG4-related diseases affecting the nervous system predominantly manifest as proliferative meningitis and hypophysitis. Studies confirm that IgG4-related proliferative meningitis may present with normal or mildly elevated cerebrospinal fluid (CSF) cell counts and normal or mildly elevated protein levels (11). The cerebrospinal fluid findings in this study are consistent with the aforementioned conclusions, showing normal cerebrospinal fluid cell counts and mildly elevated protein levels. Typical pathological changes are crucial for diagnosing IgG4-related diseases, and pathological testing plays a key role in differential diagnosis, enabling the exclusion of mimicking manifestations of other diseases. Typical histopathological features of IgG4-related disease include dense plasma cell infiltration, stellate fibrosis, occlusive phlebitis, and IgG4-positive plasma cells/IgG-positive plasma cells constituting >40% of cells (3). All 16 patients in this study underwent histopathological examination, with all biopsy specimens demonstrating characteristic features of IgG4-related disease. Neuropathology biopsy is required for IgG4-related disease with peripheral neuropathy. Based on previous case reports, biopsy sites combining nerves with enlarged glands or organs may further enhance diagnostic accuracy in IgG4-related disease with peripheral nerve involvement. In summary, serum IgG4 level measurement and biopsy are essential components for diagnosing IgG4-related peripheral neuropathy.

In regards to treatment, glucocorticoids remain the first-line therapy for IgG4-related disease. The currently recommended initial dose is moderate-dose prednisone (30–40 mg/day), maintained for 2–4 weeks before gradual tapering to the lowest effective dose, followed by a specific maintenance period (the exact duration remains controversial) (12). However, the initial dose of different glucocorticoids should be adjusted based on patient age, body weight, organ involvement, and disease severity. A review of IgG4-related peripheral neuropathy recommends a regimen combining prednisone (40–60 mg/day, followed by tapering and discontinuation over 2–3 months) with rituximab (intravenous 1 g administered in two doses 2 weeks apart, repeated every 6 months) (3). Among the cases reviewed in this paper, except for 2 cases where treatment details were unavailable, the remaining 14 cases received intravenous or oral methylprednisolone therapy. The initial dose was predominantly 0.5–0.6 kg/day (equivalent to 30–40 mg/day), maintained for 3–4 weeks before tapering. After reducing to 5 mg, long-term maintenance therapy was initiated. Two cases experienced symptom recurrence after discontinuing oral methylprednisolone (Case 4, Case 8), ultimately resolving with reinitiation of low-dose steroid maintenance. In three cases with prominent neurological symptoms, immunosuppressants were added: methotrexate, mycophenolate mofetil, or rituximab. In summary, intravenous or oral corticosteroids demonstrate favorable efficacy for IgG4-related peripheral neuropathy. Gradual tapering followed by long-term low-dose oral maintenance effectively prevents symptom recurrence. Immunosuppressive agents should be selected based on individual patient circumstances.

The present study has limitations. First, the patient in this case report did not undergo a pancreatic biopsy concurrently with the Submandibular Gland biopsy, which may have resulted in the failure to obtain additional evidence of IgG4-related disease. Second, although the patient exhibited bilateral symptoms, we chose to biopsy the more symptomatic left peroneal nerve, which may have led to differences in the pathological findings. Finally, the retrospective data consist solely of case reports, raising the possibility of racial and gender disparities. The extrapolation of conclusions requires validation through further prospective cohort studies.

## 5 Conclusion

In conclusion, our study demonstrates that neurologists should remain vigilant for rare conditions such as IgG4-related peripheral neuropathy when encountering peripheral neuropathy accompanied by multiorgan enlargement. When considering atypical causes of peripheral neuropathy, IgG4-related disease should be included in the differential diagnosis. Multiorgan imaging studies, pathological biopsy, and serum IgG4 level screening can aid in confirming the diagnosis.

## Data Availability

The original contributions presented in the study are included in the article/Supplementary material, further inquiries can be directed to the corresponding author.
