# Supplementary material for: When the nerve speaks first: IgG4-related disease unmasked by peripheral neuropathy
Source: Front Neurol. 2026 Feb 19;17:1776740. doi: 10.3389/fneur.2026.1776740 (PMC12960157; doi:10.3389/fneur.2026.1776740)
Supplement: Supplementary file 1 [file Supplementary_file_1.docx]

| **Data collection** | | | | | | | | | | | | |
| --- | --- | --- | --- | --- | --- | --- | --- | --- | --- | --- | --- | --- |
| No. | Age onset | Sex | Index  neurosymptom | Neuro onset to  system | serum IgG4 (mg/dL) | Organ enlargement | Nerve Biopsy  site | Path IgG4  ratio | Path  fibrosis | EMG  pattern | Steroid  Dose  initial | Treatment response |
|  |  |  |  |  |  |  |  |  |  |  |  |  |
|  |  |  |  |  |  |  |  |  |  |  |  |  |
|  |  |  |  |  |  |  |  |  |  |  |  |  |
|  |  |  |  |  |  |  |  |  |  |  |  |  |
|  |  |  |  |  |  |  |  |  |  |  |  |  |
|  |  |  |  |  |  |  |  |  |  |  |  |  |
|  |  |  |  |  |  |  |  |  |  |  |  |  |
|  |  |  |  |  |  |  |  |  |  |  |  |  |
|  |  |  |  |  |  |  |  |  |  |  |  |  |
|  |  |  |  |  |  |  |  |  |  |  |  |  |
|  |  |  |  |  |  |  |  |  |  |  |  |  |
|  |  |  |  |  |  |  |  |  |  |  |  |  |
