# Supplementary material for: When the nerve speaks first: IgG4-related disease unmasked by peripheral neuropathy
Source: Front Neurol. 2026 Feb 19;17:1776740. doi: 10.3389/fneur.2026.1776740 (PMC12960157; doi:10.3389/fneur.2026.1776740)
Supplement: Supplementary file 2 [file Table_1.docx]

| Supplementary Table 1 Pathological findings of participants | | | | |
| --- | --- | --- | --- | --- |
| No. | Age/Sex | Biopsy site | Pathological findings | references |
| 1 | 69/F | Kidney | histopathological examination showed fibrosis accompanied by considerable infiltration of plasma cells, and IgG and IgG4 immunohistochemical staining revealed an IgG4/IgG ratio of 64.9 %. | (15) |
| 2 | 81/F | lip, submandibular  lymph node | The lip and submandibular lymph node biopsies revealed lymphoplasmacytic infiltration with an IgG4-/IgG-positive  cell ratio of >70%. | (16) |
| 3 | 69/M | left liver,Sural nerve | liver biopsy showed storiform fibrosis and lymphoplasmacytic infiltration with IgG4-positive cells( Lymphoplasmacytic infiltration with IgG4-positive cellswas evident, accounting for >45% of the IgG positive cells, with more than 50-60 IgG4-positive plasma cells per high-power field).Sural nerve biopsy detected vascular occlusion, recanalization, and epineural fibrosis. | (16) |
| 4 | 65/M | Pancreatitis,Sural nerve | Pancreas biopsy revealed pancreas fibrosis, and the IgG4-positive plasmocytes were more than 10 per high power field under the microscope.The sural nerve biopsy was also performed and the aggregation of inflammatory cells including some IgG4-positive plasmocytes were found in the epineurium and perivascular area, besides, obstructive vasculitis was also observed. | (17) |
| 5 | 55/F | Kidney,Sural nerve | Kidney biopsy showed storiform fibrosis and plasma cell-rich  interstitial inflammation with IgG4 immunostaining showing  clusters of IgG4-positive plasma cells.IgG4 immunohistochemical staining demonstrated a few scattered IgG4-positive plasma cells(sural nerve). | (18) |
| 6 | 55/M | Skin, Sural nerve | Skin biopsy findings:Fibrosis and infiltration of inflammatory cells were observed in the subcutaneous tissue in addition to numerous IgG4-positive plasma cells.Sural nerve biopsy findings:More than 10 IgG4-positive plasma cells exist in the epineurium . | (19) |
| 7 | 56/M | Skin, Sural nerve | Skin biopsy and sural nerve biopsy revealed  reactive lymphoid hyperplasia with increased  IgG4-positive cells. | (20) |
| 8 | 82/M | lacrimal gland | IgG-positive plasma cells on the lymphoid  follicles were visualized by enzyme immunostaining | (21) |
| 9 | 77/M | kidney | Biopsies of the kidney showed membranous  nephropathy with numerous IgG4-positive  plasma cells. | (22) |
| 10 | 73/M | Lung mass,Sural nerve, perinephric stranding | Biopsy of the lung mass demonstrated fibrosis with patchy in flammation and mild increase in IgG4 cells.biopsy of the perinephric stranding was pursued which demonstrated fibroadipose tis sue with mild chronic inflammation and mild increase in IgG4. | (10) |
| 11 | 31/M | Aortic arch mass | Histologic analysis of the thoracoscopic biopsy specimen of the mass at the aortic arch demonstrated diffuse infiltration of lymphoplasmacytes and marked fibrosis. immunostaining for IgG4 revealed many IgG4-positive plasma cells within the lesion. | (9) |
| 12 | 63/M | submandibular gland, Sural nerve | Biopsy of the right submandibular gland was suggestive of IgG4/IgG positive plasma cells >50%.Immunohistochemistry was positive for CD 68, CD 20 and CD 138 of sural nerve. | present case |
| 13 | 55/M | orbital muscles | The orbital biopsy showed an increased number of IgG4-positive plasma cells constituting at least 50% of total IgG plasma cells and more than 30 IgG4+ plasma cells per high-power field. | (1) |
| 14 | 64/M | Mediastinal lymph node | Histologic findings of the mediastinal lymph node. Abundant CD138-positive plasma cell infiltration is observed. The ratio of IgG4-positive plasma cells/IgG-positive cells is approximately 80%. | (23) |
| 15 | 74/M | Pleural | Pleural biopsy showed lymphoid and lymphoplasmacytic collections along with areas of infiltration by eosinophils and histiocytes. Immunohistochemistry showed an increase in IgG4 staining with an IgG4 to IgG ratio of approximately 30%. | (25) |
| 16 | 51/M | S1 nerve root, perirenal tissue | S1 nerve root biopsy showed the presence of aggregated lymphocytes, surrounded by diffusely growing fibrous tissue, and was positive for CD3 and CD20.Perirenal lesion samples revealed the presence of lymphocytes, fibrous tissue, and plasma cells. The ratio of IgG4-positive plasma  cells to IgG-positive cells exceeded 0.5. | (24) |
|  |  |  |  |  |
